# Effect evaluation of outpatient long-term video EEGs for people with seizure disorders – study protocol of the ALVEEG project: a randomized controlled trial in Germany

**DOI:** 10.1186/s12913-024-11076-y

**Published:** 2024-08-27

**Authors:** Pauline Sarah Münchenberg, Ricarda Sophia Schulz, Kerstin Wainwright, Imke Mayer, Martin Holtkamp, Christian Meisel, Tobias Kurth, the ALVEEG Consortium, Bernd Vorderwülbecke, Mirja Steinbrenner, Matthias Endres, Claudia Gorski, Fabian Prasser, Angela Kaindl, Bernhard Weschke, Cornelia Potratz, Pascal Fenske, Felix von Podewils, Astrid Bertsche, Sarah Mai Viebahn, Bernadette Gaida, Norbert Utzig, Juliane Schulz, Thomas Mayer, Peter Hopp, Nils Holert, Miriam Wienecke, Georg Leonhardt, Peggy Müller, Petra Knobelsdorf, Antke Wolter, Anne Klinker, Mara Brandebusemeyer, Uwe Nussbaum, Jannis Seemann

**Affiliations:** 1 Institute of Public Health, Charité – Universitätsmedizin Berlin, Berlin, Germany; 2 Department of Neurology with Experimental Neurology, Charité – Universitätsmedizin Berlin, Berlin, Germany; 3 Center for Stroke Research Berlin, Charité – Universitätsmedizin Berlin, Berlin, Germany; 4 Berlin Institute of Health, Charité – Universitätsmedizin Berlin, Berlin, Berlin, Germany; 5 NeuroCure Cluster of Excellence, Charité – Universitätsmedizin Berlin, Berlin, Germany; 6 Bernstein Center for Computational Neuroscience, Berlin, Germany

**Keywords:** Seizure disorders, Epilepsy, Healthcare management, Outpatient long-term video EEGs, Statutory health insurance, Randomized controlled trial, Equivalence trial

## Abstract

**Background:**

Epilepsy and other seizure disorders account for a high disease burden in Germany. As a timely diagnosis and accurate treatment are crucial, improving the management of these disorders is important. Outside of Germany, outpatient long-term video EEGs (ALVEEGs) have demonstrated the potential to support the diagnosis and management of epilepsy and other seizure disorders. This study aims to evaluate the implementation of ALVEEGs as a new diagnostic pathway in eastern parts of Germany to diagnose epilepsy and other seizure disorders and to assess if ALVEEGs are equally effective as the current inpatient-monitoring gold standard, which is currently only available at a limited number of specialized centers in Germany.

**Methods:**

ALVEEG is a prospective, multicenter, randomized controlled equivalence trial, involving five epilepsy centers in the eastern states of Germany. Patients will be randomized into either intervention (IG) or control group (CG), using a permuted block randomization. The sample size targeted is 688 patients, continuously recruited over the trial. The IG will complete an ALVEEG in a home setting, including getting access to a smartphone app to document seizure activity. The CG will receive care as usual, i.e., inpatient long-term video-EEG monitoring. The primary outcome is the proportion of clinical questions being solved in the IG compared to the CG. Secondary outcomes include hospital stays, time until video EEG, time until diagnosis and result discussion, patients’ health status, quality of life and health competence, and number and form of epilepsy-related events and epileptiform activity. Alongside the trial, a process implementation and health economic evaluation will be conducted.

**Discussion:**

The extensive evaluation of this study, including an implementation and health economic evaluation, will provide valuable information for health policy decision-makers to optimize future delivery of neurological care to patients affected by epilepsy and other seizure disorders and on the uptake of ALVEEG into standard care in Germany.

**Trial registration:**

German Clinical Trials Register (DRKS00032220), date registered: December 11, 2023.

**Supplementary Information:**

The online version contains supplementary material available at 10.1186/s12913-024-11076-y.

## Introduction

### Background and rationale

Epilepsy is one of the most common chronic neurological conditions worldwide, imposing a high burden of disease on people affected and on society [1]. In Germany, around 500,000 people are affected by epilepsy [2]. An accurate diagnosis of epilepsy and other seizure disorders, such as psychogenic non-epileptic seizures or syncopes, is crucial to avoid misdiagnoses and to create a correct treatment plan, leading to an improved quality of life (QoL) for people affected and preventing fatal seizures [3].

Therefore, equipping healthcare systems with the right tools to make a timely diagnosis is important to avoid a lack of healthcare services, especially in rural areas [4]. The current gold standard for diagnosing epilepsy and other seizure disorders is an inpatient long-term video-electroencephalogram (EEG) monitoring (VEM). However, inpatient monitoring is only available at limited specialized centers and is associated with significant time and work resources [5]. Further, there are still fewer neurologists and pediatric neurologists in the rural eastern parts of Germany, reflected by a higher number of epilepsy patients per neurologist, leading to a significant waiting time for inpatient long-term video EEGs [6]. To address these challenges, outpatient long-term video EEGs (ALVEEGs) have already been established in other countries [7]. The development of a new portable sensor technology, enhanced by innovative automated data analyses supported by artificial intelligence (AI), allows ALVEEGs in the patient’s home environment. This advancement facilitates faster and more accurate diagnoses in this setting. By aligning with the treatment gold standard of care, it helps preventing misdiagnoses and inappropriate treatment plans, thereby improving QoL of people affected by epilepsy and other seizure disorders [8].

As shown in a single-center feasibility study in Germany, ALVEEGs were well accepted by patients and clinicians. They may offer an equivalent way of diagnosing epilepsy and other seizure disorders in Germany [8].

The here presented study, funded by the German Innovations Fund of the Federal Joint Committee (G-BA), aims to evaluate if ALVEEGs in an outpatient setting as diagnostic pathways are equally effective as the current inpatient gold standard of VEM to diagnose epilepsy and other seizure disorders. If successful, barriers in diagnosis and treatment care may be resolved, and patients in Germany affected by these disorders may have better and more rapid access to adequate diagnostics and subsequent treatment initiation or optimization.

### Study aim and objective

The aim of ALVEEG is to improve and optimize care of patients experiencing epilepsy and other seizure disorders in Germany by translating case and care management into the patient’s home environment by introducing a new standardized way of diagnosing epilepsy and optimizing disease management in the eastern states of Germany. The objectives of the evaluation are to (1) analyze the effects of ALVEEG on patient care compared to the current standard inpatient VEM, assessing patients who have had at least one seizure in the last 12 months or a diagnosis of epilepsy, (2) observe the implementation of project activities, and (3) conduct a health economic evaluation.

## Methods

The presented study protocol follows the SPIRIT reporting standards [9] and is aligned with the reporting guidelines for randomized trials, i.e., CONSORT [10] and the TIDieR checklist [11] for intervention description and replication.

### Trial design

ALVEEG is a prospective, multicenter, randomized controlled trial (RCT) with a project duration of 4 years. The study is designed as an equivalence trial, with a trial duration of 27 months and continuous patient recruitment.

Patients assigned to the control group (CG) receive care as usual, i.e., inpatient VEM, while those randomized to the intervention group (IG) undergo outpatient ALVEEG.

### Study setting and eligibility criteria epilepsy centers

ALVEEG is conducted in cooperation with five epilepsy centers in the eastern states of Germany (Berlin, Brandenburg, Mecklenburg-West Pomerania, and Saxony). Eligible epilepsy centers had to (1) be led by neurologists/neuropediatrics/epileptologists, (2) meet the technical and spatial requirements for conducting long-term inpatient video EEGs, and (3) treat patients who are part of the statutory health system. Epilepsy centers were excluded if they (1) were located outside the catchment area, (2) did not meet the technical and spatial requirements, or (3) did not treat patients who are part of the statutory health system.

The following five epilepsy centers participate in the study: Charité – Universitätsmedizin Berlin, Epilepsy-Center Berlin-Brandenburg; Charité – Universitätsmedizin Berlin, Germany Epilepsy-Center for Children and Adolescents; University Hospital Greifswald; Epilepsy-Center Kleinwachau; and University Hospital Dresden.

Participating statutory health insurances are BARMER, DAK-Gesundheit and Techniker Krankenkasse (TK).

### Eligibility criteria and patient recruitment

To be eligible to participate in ALVEEG, patients have to (1) have had at least one seizure in the last 12 months or a diagnosis of epilepsy, (2) have an indication 1–4 from OPS 1-210, (3) live in a household with access to at least 4G or faster, (4) be part of the German statutory health insurance system, and (5) present themselves at least at one of the participating epilepsy centers. Patients can only be included if they or their legal representative gave written informed consent. Given the open cohort design of the trial, patients will be continuously recruited by the participating epilepsy centers over the trial duration of 27 months.

Patients are excluded if they (1) have an epilepsy surgery indication (indication 5 from OPS 1-210), (2) present themselves with additional psychosocial needs such as consultation on occupational or driving issues, (3) are privately insured, self-paying patients, or (4) if they or their legal representative does not give informed consent.

In patients with an epilepsy surgery indication, ictal events require some acute neuropsychological testing performed by medical technicians. Therefore, these patients are excluded for medical reasons. Further, patients must have had at least one seizure in the last 12 months or an epilepsy diagnosis to use disease-specific questionnaires during the trial, e.g., the Quality Of Life In Epilepsy (QOLIE-10) questionnaire, to get better insights on seizures relevant for the included population.

### Intervention

The IG will complete an ALVEEG at home, comprising a portable EEG- and electrocardiogram (ECG)-recorder and a mobile data station, including a camera and audio recorder. The ambulatory monitoring system is provided by a medical device company that develops, manufactures, and commercializes diagnostic technologies for brain monitoring applications. EEG data will be reviewed using a review and analysis software, which offers computer-assisted reviews of EEGs, supported by AI. Patients will also get access to the smartphone app Helpilepsy [12], a digital diary for the documentation of seizures and related factors throughout the study. Alternatively, patients can also choose to use a paper diary for documentation.

There will be four appointments in the IG at the participating centers. During the first appointment, the EEG and ECG electrodes will be fitted on the patient’s head and chest to measure brain and heart activity and are connected with a portable amplifier. The ALVEEG will take place in the patient’s home environment, recording EEG, ECG, video, and audio data for up to 6 days. Patients will be monitored throughout the study for possible adverse events (AE; e.g., skin irritations or mild rashes) that might occur, given the nature of the intervention. All AE will be recorded to inform future guidelines and use of ALVEEGs. Patients are also asked to document seizures via the app or the paper diary throughout the monitoring. After completing their ALVEEG, patients can either remove the electrodes themselves at home or they are removed in the clinic when patients return all devices at their second appointment. During the third appointment, results of the ALVEEG will be discussed, and the patient’s therapy and treatment plan will be amended accordingly. The patient will also get a medical findings report. The fourth and final appointment will take place three months after completing the VEM for the last follow-up and the final collection of study-related patient-reported outcome measures (PROMs).

### Study outcomes

The outcomes of the study refer to the healthcare system (macro level), the institutional level (meso level) as well as the individual patient (micro level).

The primary outcome of ALVEEG is the proportion of clinical questions being solved in the IG compared to the CG. A clinical question is considered as solved if (1) a specified diagnosis is needed and provided via long-term video EEG, (2) treatment management needs to be defined and is provided via long-term video EEG, or (3) a specified diagnosis is needed, and treatment management needs to be defined according to findings of the long-term video EEG.

Secondary outcomes (see Table 1), include on the macro level: (1) number of hospital stays, (2) length of hospital stays, (3) inability to work (in days), and (4) number of video EEGs; on the meso level: (5) duration of waiting period for a video EEG (in days), (6) duration of waiting period for diagnosis and result discussion, (7) duration of diagnosis identification using AI, and (8) duration of long-term video EEG; and on the micro level: (9) number of diagnosis specifications, definition of treatment management and diagnosis specifications combined with treatment management, (10) number and form of epilepsy-related events and epileptiform activity, (11) QoL related to epilepsy, (12) QoL, (13) health status, (14) health competence, and (15) contentment, acceptance, and AE.

**Table 1:** Secondary outcomes, time points, and measurement instruments

Outcome	Timepoint	Measurement Instrument
Number of diagnosis specifications, treatment management, diagnosis specification & treatment management (IC-CG)	tP1	Study data base
Number and type of epilepsy-related events or epileptiform activity (ictal, interictal, only video as quality indicator) *(IC-CG)*	*tD2*	Study data base
Waiting time for video EEG *(IC-CG)*	*t0-tSens*	Study data base
Duration until discussion of diagnosis/results *(IC-CG)*	*t0-tP1*	Study data base
Epilepsy-related quality of life *(IC-CG)*	*t0,tP2*	QOLIE-1 (PROM) via tablet, study data base
Quality of life *(IC-CG)*	*t0,tP2*	EQ-5D-5L (PROM) via tablet, study data base
Health status *(IC-CG)*	*t0,tP2*	EQ-5D-5L VAS (PROM) via tablet, study data base
Health competence/seizure awareness *(IC-CG)*	*t0,tP2*	Subscale PESOS (PROM) via tablet, study data base

*t0 *at baseline*, tD2 *after diagnosis compilation by the neurologist*, tP1 *appointment to discuss medical findings (patient and neurologist)*, tP2 *3-month follow up appointment (patient and neurologist)*, tSens *time point of sensor/electrodes placement

### Participant timeline

Randomization of patients into either the IG or CG will take place in the participating epilepsy centers at baseline (*t0*), after patients have given their informed consent. The treating neurologist will be informed about the patient’s group assignment, and all included patients are asked to complete REDCap (Research Electronic Data Capture) surveys on PROMs (IG and CG: EQ-5D-5L, EQ-5D-5L (VAS), as well as the QOLIE-10 and a subscale of the PESOS questionnaire). All questionnaires will be saved and managed in REDCap electronic data capture tools, hosted at the University of Nebraska Medical Center. REDCap is a secure, web-based application designed to support data capture for research studies [13, 14].

**Fig. 1:**
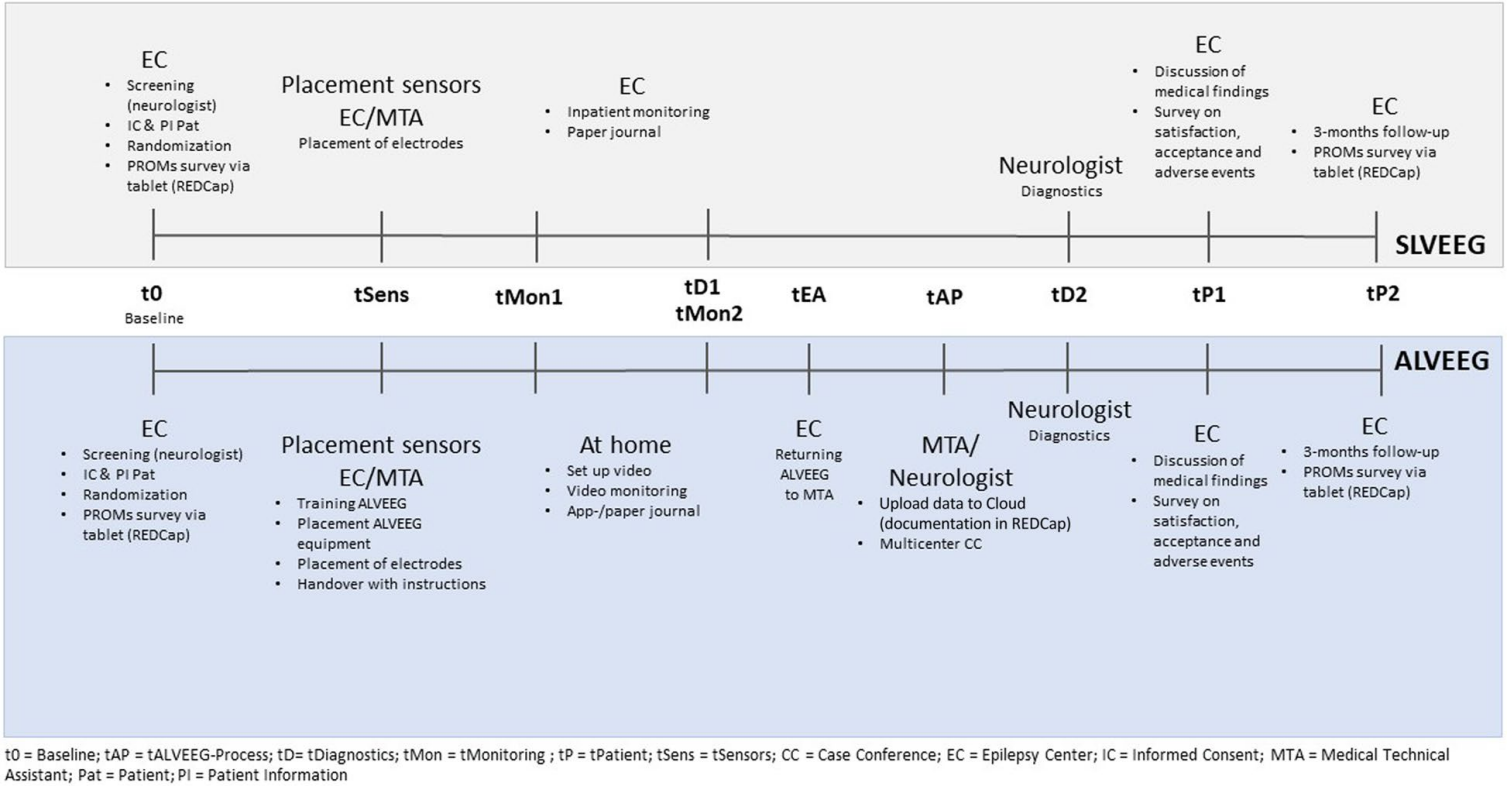
Participant timeline throughout the course of the study

After randomization, patients will receive either inpatient (CG) or outpatient (IG) long-term VEM, with patients in the IG being trained in how to use ALVEEG (*tSens*). They can also call at any time if problems occur during the monitoring at home (*tMon1-tMon2*), and there will be a check-in on the first day of ALVEEG, led by a medical-technical assistant (MTA). Secondary outcome data will be assessed for both IG and CG throughout the study at different assessment time points (see Fig. 1). After the ALVEEG is completed (*tMon2*), patients in the IG will return the equipment to the hospital (*tEA*). The ALVEEG data will be uploaded into the cloud provided by the medical device company and documented in REDCap (*tAP*). The patient’s practitioner will also get access to the monitoring results, which are evaluated by the neurologist for patients in the IG and CG (*tD2*). After the evaluation of the monitoring is completed, the neurologist will discuss the results and care and treatment plan with the patient in both IG and CG (*tP1*). Patients will additionally answer a survey on their acceptance, contentment, and AE during ALVEEG. The follow-up assessment will take place three months after the discussion of medical findings, with patients in both groups answering the PROM questions in REDCap again (*tP2*).

### Data management, collection, and quality control

All surveys for neurologists, MTAs and patients will be conducted electronically via a tablet. The answers will be documented in REDCap, constituting the ALVEEG study database.

REDCap will be programmed using automatic plausibility checks and mandatory fields for data entry. Measures to inquire about missing data by the study personnel will be instituted.

Regular data consistency and plausibility checks will take place in cooperation with the evaluation team, which consequently will contribute to a better quality of documentation in the epilepsy centers. Technical training of the epilepsy centers and study personnel regarding the use of ALVEEG and its AI-supported diagnosis, as well as the documentation in REDCap, will take place prior to the start of the intervention. All questionnaires that were developed specifically for this study were piloted before the start of the intervention.

A data protection concept for the study has been accepted by the data protection officers in charge at the Charité – Universitätsmedizin Berlin. It comprises guidelines with respect to collecting data, data management, transfer, processing, and storage. Data originating from clinical care process and claims data of the participating health insurance companies (primary and secondary data) will be securely transferred from a data integration center (composited of the independent data trust agency (“Treuhandstelle”) at the Charité – Universitätsmedizin Berlin and a team for medical informatics at the Berlin Institute of Health) to the independent evaluating institution at the Charité – Universitätsmedizin Berlin where they will get analyzed and findings will be provided to the studies funding agency.

### Statistical methods: sample size, recruitment and power calculations

The sample size calculation for the presented equivalence trial is based on the primary endpoint. After consultation with the clinical consortium partners, the proportion of resolved clinical cases in the CG is assumed to be 0.8 (80%). We expect to observe an equivalent rate of treatment successes in the IG, whereby (clinical) equivalence is defined within a margin of +/- 15% deviation from the rate in the CG. This corresponds to an equivalence interval of [0.68–0.92] ([68 − 92%]) for the proportion of clinical questions being solved. This margin is based on assumptions made by clinical experts within the consortium and is further supported by the results of international studies on the subject [15].

Based on a significance level of 0.05 and a power of (1-β) = 90%, applying two proportions z-test results in a sample size of 482 participants. Considering an expected dropout rate of about 30%, the sample size of the study amounts to 688 participants. In order to achieve this sample, an average of approximately five patients must be recruited per center and month.

For the secondary endpoints, a power of 0.83 was computed using a two-sided t-test for 550 patients, taking into account the latency period of the routine data from the participating health insurance funds.

According to estimations by the consortium partners and the involved health insurance companies, it is assumed that the aspired sample size can be achieved for all endpoints.

### Sequence generation and randomization procedure

For ALVEEG, a permuted block randomization with block sizes of either 2 or 4 patients [16] was performed at the patient level prior to the start of the trial by an independent data analyst of the evaluation team. Each participating center was assigned its own randomization list.

Given the nature of the trial design, blinding the treating neurologist is impossible. After the patients or their legal representatives have given informed consent and the neurologist has discussed their further treatment strategy with them, patients will be aware of their group allocation.

### Statistical methods

The primary analysis has the aim to evaluate the effect of ALVEEG based on the predefined primary endpoint, the proportion of cases being solved by the video EEG. Two one-sided z-tests will be performed, and a confidence interval will be determined based on the observed difference between IG and CG and interpreted using the predefined equivalence interval, analyzing the proportion of clinical questions being solved in the IG compared to the CG [17]. Point estimates of the intervention effect, together with the corresponding 95% confidence intervals, will be reported on the absolute and relative scale.

For the planned descriptive statistical analyses, mean values (standard deviation) for continuous normally distributed data, medians and interquartile ranges for non-normally distributed continuous data, and the proportions of frequencies for categorical data will be presented.

Depending on the relevant variables, various statistical test procedures and multivariable techniques will be applied in the context of secondary and sensitivity analyses to test the hypotheses within the context of inferential statistics. In principle, the inferential data analyses focus on differences between the IG and CG. For example, to test the difference in duration in days (e.g., waiting time until video EEG or length of in-hospital stay), we will perform a t-test for normal distributions or the Wilcoxson rank sum test for non-normally distributed data.

The entire statistical data analysis will be performed using the statistical software *R* [18] *v4.2.1* (or higher).

Prior to data lock and the actual extraction of the data, a detailed statistical analysis plan, including additional information on ancillary analyses and the handling of missing data, will be developed.

### Evaluation of the implementation of ALVEEG

To evaluate the implementation of ALVEEG, its delivery, further implementation and its generalizability, we will use the RE-AIM framework [19]. The RE-AIM framework facilitates assessing the benefits of innovative forms of care, while taking into account their successful implementation [20].

Therefore, the number of approached patients compared to those actually participating is measured to assess whether the intervention reaches the intended target population (REACH). The number of achieved treatment successes will also be recorded and evaluated accordingly (EFFECTIVENESS). In addition, we will examine the extent to which the included study sites participated in the intervention (ADOPTION) and the implementation of intervention activities according to protocol (IMPLEMENTATION). Furthermore, we will investigate the degree to which the intervention can be sustainably integrated into routine processes (MAINTENANCE).

### Health economic evaluation

A health economic evaluation will be carried out alongside the trial, investigating whether the intervention is cost effective compared to standard care. The evaluation will be performed from a statutory health insurance perspective and will comprise a cost-utility as well as additional cost-effectiveness analyses. The outcome to be considered for the cost-utility analysis will be quality-adjusted life years (QALYs), calculated by data obtained from the EQ-5D-5L questionnaire under applying German population weights [21].

Additional emerging costs with relevance to the intervention will be considered compared to standard care (e.g., ALVEEG devices, staff education, etc.), as well as costs per patient stemming from health insurance data (e.g., number of hospitalizations, medications, travel expenses, etc.).

## Discussion

The shortage of inpatient VEM capacities for patients with epilepsy and other seizure disorders in Germany, in addition to the artificial environment of VEMs taking place in hospitals instead of the patient’s usual surroundings, requires better care and access for this population - especially in rural areas, where resources and neurological specialists are limited. As shown in previous studies from other countries, ALVEEGs allow a faster and more effective diagnosis and management of epilepsy and other seizure disorders [7, 8].

We expect that results from this study will deliver insightful evidence on the effectiveness of ALVEEGs in diagnosis and management of epilepsy and other seizure disorders compared to the current inpatient gold standard - not only on the patient and clinical level but also with respect to the German statutory health care system. Further, we will be able to provide a more nuanced understanding of the intervention and its impact, as well as cost-utility and cost-effectiveness results, as we are evaluating different endpoints, while taking into account multiple outcomes and intervention-associated costs.

Nevertheless, there are some limitations to our study. As we collect data from a real-world clinical care setting, we expect missing data to occur, especially when it comes to self-reported information. Being aware of this, we will address this issue through the elaboration of statistical strategies to account for missing values for the different endpoints to be assessed. Further, as blinding of the treating neurologist and data analyst is not possible due to the nature of the trial design, potential bias cannot be ruled out. However, we do not expect any major impact, i.e., bias on the effects to be analyzed.

Finally, claims data from German statutory health insurances are subject to a waiting period for up to 9 months. Therefore, it will not be possible to analyze claims data from all included patients. Further, we will only be able to analyze claims data for patients who are part of the participating three statutory health insurances: BARMER, DAK-Gesundheit and TK. Although in total there are 96 statutory health insurances in Germany, the insurance companies participating in this trial are part of the biggest German statutory health insurances [22]. Hence, we believe that patients for whom claims data will be available are representative of the entire ALVEEG study population.

One major strength of the study is its pragmatic nature, allowing to generate real-world evidence on the use of ALVEEGs in clinical care processes as a new diagnostic pathway in epilepsy centers in Germany. Although we do not expect any possible harm for participating patients, we made sure that potentially occurring AE during the trial will be continuously monitored and patients will have access to professional support throughout the trial.

The evaluation of this study will provide valuable information for decision makers to optimize future delivery of neurological care to patients affected by epilepsy and other seizure disorders, and to guide a possible scale-up of ALVEEGs in Germany. Further, conducting an implementation and health economic evaluation alongside this trial will provide essential information for key stakeholders in the German healthcare system.

### Trial status

Patient recruitment started in April 2024. In June 2026, the last patient will be recruited. Results are expected in 2027.

### Supplementary Information

Supplementary Material 1.

## Data Availability

No datasets were generated or analyzed during the current study.

## Abbreviations

AE: Adverse events
AI: Artificial intelligence
ALVEEGs: Outpatient long-term video EEG
CG: Control group
ECG: Electrocardiogram
EEG: Electroencephalogram
EQ-5D-5L: European quality of life questionnaire – 5 dimensions
G-BA: German Innovations Fund of the Federal Joint Committee
ICER: Incremental cost-effectiveness ratio
IG: Intervention group
MTA: Medical-technical assistant
OPS: Operation and procedure classification system
PESOS: Performance, Socio-demographic aspects, Subjective evaluation Fragebogen
PROMs: Patient reported outcome measures
QALYs: Quality-adjusted life years
QoL: Quality of life
QOLIE-10: Quality of life in epilepsy
RCT: Randomized controlled trial
REDCap: Research Electronic Data Capture
TK: Techniker Krankenkasse
VAS: Visual analogue scale
VEM: Video-EEG monitoring
