# Supplementary material for: Effect evaluation of outpatient long-term video EEGs for people with seizure disorders – study protocol of the ALVEEG project: a randomized controlled trial in Germany
Source: BMC Health Serv Res. 2024 Aug 27;24:994. doi: 10.1186/s12913-024-11076-y (PMC11348661; doi:10.1186/s12913-024-11076-y)
Supplement: Supplementary file 1 — Supplementary Material 1. [file 12913_2024_11076_MOESM1_ESM.pdf]

ALVEEG Study Protocol: Additional File 1

The ALVEEG Consortium:

Pauline Sarah Münchenberg[1], Ricarda Sophia Schulz[1], Tobias Kurth[1]

Christian Meisel[2,3,4,5,6♣], Martin Holtkamp[2], Bernd Vorderwülbecke[2], Mirja Steinbrenner[2], Matthias Endres[2], Claudia Gorski[2]

Fabian Prasser[4]

Angela Kaindl[7], Bernhard Weschke[7], Cornelia Potratz[7], Pascal Fenske[7]

Felix von Podewils[8], Astrid Bertsche[8], Sarah Mai Viebahn[8], Bernadette Gaida[8], Norbert Utzig[8], Juliane Schulz[8]

Thomas Mayer[9], Peter Hopp[9], Nils Holert[9], Miriam Wienecke[9]

Georg Leonhardt[10]

Peggy Müller[11], Petra Knobelsdorf[11], Antke Wolter[11]

Anne Klinker[12], Mara Brandebusemeyer[12]

Uwe Nussbaum[13], Jannis Seemann[13]

Affiliations:

[1] Institute of Public Health, Charité – Universitätsmedizin Berlin, Berlin, Germany

[2] Department of Neurology with Experimental Neurology, Charité – Universitätsmedizin Berlin, Berlin, Germany

[3] Berlin Institute of Health, Berlin, Germany

[4] NeuroCure Cluster of Excellence, Charité – Universitätsmedizin Berlin, Berlin, Germany

[5] Bernstein Center for Computational Neuroscience, Berlin, Germany

[6] Department of Neuropediatric Services, Charité – Universitätsmedizin Berlin, Berlin, Germany

[7] University Hospital Greifswald

[8] Institute of Public Health, Charité – Universitätsmedizin Berlin, Berlin, Germany

[9] Epilepsy-Center Kleinwachau

[10] University Hospital Dresden

[11] DAK-Gesundheit, Hamburg, Germany

[12] Techniker Krankenkasse, Hamburg, Germany

[13] BARMER, Berlin, Germany

♣ Nominated Consortium representative
